# Network indicators of cultural resilience to anthropogenic removals in animal societies

**DOI:** 10.1098/rstb.2024.0144

**Published:** 2025-05-01

**Authors:** Amelia C. Meier, Nicolas Restrepo Ochoa, Anna E. Nordseth, Molly Copeland, Vivienne Foroughirad, Janet Mann, George Wittemyer, Jennifer E. Smith

**Affiliations:** ^1^ University of Hawai'i at Manoa Hawaii Institute of Marine Biology, Kaneohe, HI 96744, USA; ^2^ Nicholas School of the Environment, Duke University, Durham, NC 27710, USA; ^3^ Department of Anthropology, University of California Davis, Davis, CA 95616, USA; ^4^ Department of Sociology, University of Notre Dame, Notre Dame, IN 46556, USA; ^5^ Department of Biology, Georgetown University, Washington, DC 20057, USA; ^6^ Department of Marine Biology, Texas A&M University at Galveston, Galveston, TX 77553, USA; ^7^ Department of Psychology, Georgetown University, Washington, DC 20057, USA; ^8^ Department of Fish, Wildlife, Conservation Biology, Colorado State University, Fort Collins, CO 80523, USA; ^9^ Save The Elephants, Nairobi, Kenya; ^10^ Biology Department, University of Wisconsin-Eau Claire, Eau Claire, WI 54702, USA

**Keywords:** conservation, culture, animal social networks, social learning, social transmission

## Abstract

Social learning, information transmission and culture play vital roles in the lives of social animals, influencing their survival, reproduction and ability to adapt to changing environments. However, the effect of anthropogenic disturbances on these processes is poorly understood in free-living animals. To investigate the impact of anthropogenic disturbance on social learning and information transmission, we simulated individual removal from contact networks derived from long-term behavioural datasets. We simulate the effects of individual removal on network efficiency and social learning for three group-living species—yellow baboons (*Papio cynocephalus*), African savanna elephants (*Loxodonta africana*) and Indo-Pacific bottlenose dolphins (*Tursiops aduncus*). We reveal how removals of key network positions reduce network efficiency. However, groups with high levels of innovation may cope with changing social network structures. These findings highlight the importance of protecting key individuals to preserve group structure and the role of innovation in possibly mitigating the fitness costs of removals. Identifying and safeguarding individuals that drive innovation can reduce a group’s susceptibility to anthropogenic threats and promote cultural resilience in social animals in a changing world. These emerging trends contribute to a growing understanding of the role of conservation interventions in protecting critical individuals in group-living animals.

This article is part of the theme issue ‘Animal culture: conservation in a changing world’.

## 1. Introduction

For many species, group living has positive fitness consequences for individuals [1–3]. One major benefit of group living is that key information about resources [4], mates [5], predators [6] and novel threats [7] may be gleaned socially through observation of, or interaction with, another group member. Social learning may also contribute to the transmission of novel behaviours, like the spread of novel foraging traditions or vocal repertoires. Socially learned foraging behaviours may propagate beyond an individual’s immediate social group and transfer to other social groups when they come into contact [8]. For example, in humpback whales innovative ‘lobtail’ feeding behaviour was spread via social transmission from a single whale [9] and new vocal repertoires can rapidly emerge from song hybridization events [10]. Socially learned behaviours may also impact the survival and reproduction of individuals or, in some cases, entire social groups [11]. These traits can be particularly influential because they can spread more rapidly within social groups than genetically inherited ones (i.e. within a single generation) [12]. And, although culture was previously assumed to be unique to human societies [13], socially learned and transmitted behaviours may contribute to group-specific, socially transmitted traditions or ‘animal culture’ [14,15].

Anthropogenic change can influence animal social dynamics. Evidence suggests that humans can reduce opportunities for social learning and horizontal transmission of information through the indirect reduction of group size associated with human presence [16] or via the direct removal of key individuals from groups [17]. For example, chimpanzee communities in areas with high human impact experienced an average behavioural diversity loss of 88% compared to those residing in low-impact areas [18]. Moreover, among sperm whales, anthropogenic noise reduces vocal communication [19]—a critical element of group cohesion [20]. In other cases, however, social learning can help group-living animals cope with or even exploit anthropogenic change. For instance, social learning promotes access to farmed foods by chimpanzees [21], the removal of fish from longlines by sperm whales [22] and opening of household waste bins by cockatoos [23]. The emergence and spread of innovations vital to social learning can promote adaptive responses to anthropogenic activities but also exacerbate human–wildlife conflicts. Human disturbances, for instance, may also alter fundamental group structures [24], which may lead to behavioural changes such as loss of vocal diversity in groups [25]. However, definitive links between group structural features and behavioural repertoires are lacking and the social mechanisms involved require further study.

Social network analysis is a powerful tool for assessing how group structure shapes the diffusion of social information and/or behavioural innovations within and across groups over time [26,27]. Network analysis identifies key individual positions, such as those with many direct connections (high degree centrality) or those that indirectly connect other individuals (high betweenness centrality; e.g. [28]). For instance, in ravens simple exposure to new behaviours does not guarantee social learning, and central individuals learn from and transmit information among affiliates more rapidly compared to highly related or ranked individuals [29]. Network analysis also describes overarching group structure through features like group density or cohesion, nested hierarchical social levels or modularity of subgroups [30]. Network characteristics provide underlying structural conditions that influence the diffusion of socially learned behaviours within social groups [31]. Removing an individual and their social connections can have cascading consequences on the distribution of social connections among remaining group members, affecting core processes within animal societies [32]. However, removal may affect transmission differently depending on a given network’s structural characteristics [33]. Building upon the knowledge that disturbances (natural or anthropogenic) [34] shape animal social structures, we simulated the consequences of human-induced removals on contact networks and, thus, the potential for social information transmission [32].

To gain insights into social learning dynamics and the potential for cultural transmission across networks in nature [35], we leveraged data from long-term research projects on three female-bonded social mammals: yellow baboons (*Papio cynocephalus*), African savanna elephants (*Loxodonta africana*) and Indo-Pacific bottlenose dolphins (*Tursiops aduncus;* hereafter baboons, elephants and dolphins). We chose these focal species due to (i) the hypothesized importance of social learning and cohesion in their social networks, (ii) their documented vulnerability to anthropogenic threats (i.e. poaching, bush meat, habitat loss, disease), and (iii) the availability of detailed long-term social data on these populations (for details, see the electronic supplementary material).

Understanding the consequences of group structural changes—particularly following the loss of individuals—on social information transmission could offer insights into species’ resilience to anthropogenic disturbance. Here, we investigate how social disruption affects group structure by simulating removal of individuals of known ages and evaluating its impact on inferred innovation rates. We first ask: (i) What are the defining structural features of each network? With these metrics, we identify key individuals in social groups and ask: (ii) How efficiently can each group transmit social information (based on contact networks) across members after key individuals are removed? and (iii) What is each group’s ability to solve problems after individuals are removed?

## 2. Methods

### (a) Empirical study systems for characterizing social networks

We used observational behavioural data for recognizable individuals belonging to known age categories from three long-term field studies to compare the influence of group structure on contact networks as a proxy for social learning (electronic supplementary material, table S1).

#### (i) Amboseli Baboon Research Project in Kenya

**Figure 1.**
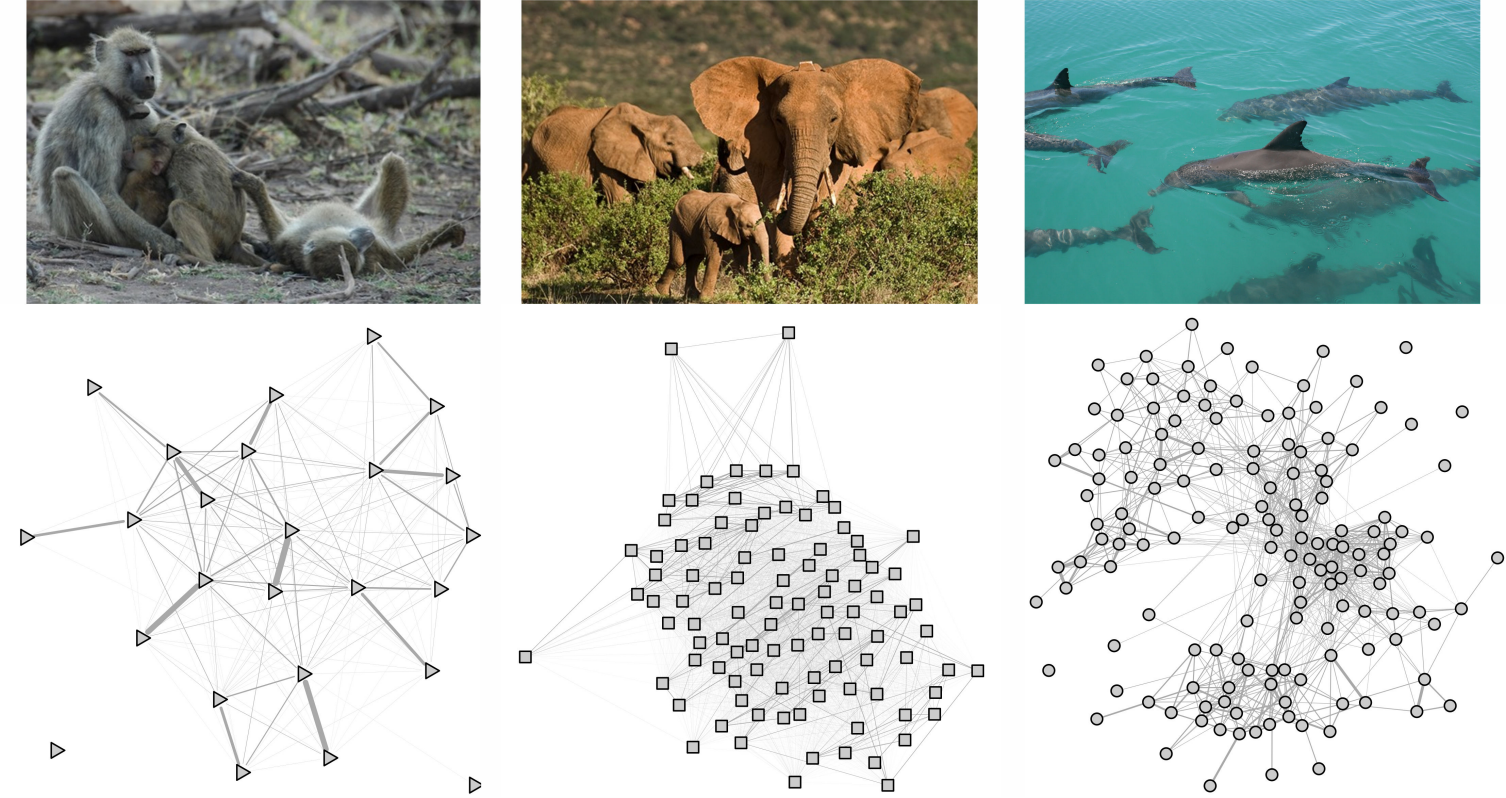
Examples of social networks comprised of females from a single wave for each of the focal species: baboons (triangles), elephants (squares) and dolphins (circles). Nodes represent individuals in the network and edges represent ties between individuals. Line darkness represents the strength of pair-wise connections. Photographs reproduced with permission from the Amboseli Baboon, Samburu Elephant, and Shark Bay Dolphin Research Projects.

East African baboons are highly cohesive social primates distributed across eastern and central Africa [36]. Their groups (troops) are structured by linear dominance hierarchies and range in size from less than 10 to nearly 200 individuals [37]. Social connections among females directly influence fitness [1]. We used data on 55 females in one social group, collected by members of the Amboseli Baboon Research Project between 1997 and 2001 (see [38] and its data, and figure 1). Group size during this period of study had a mean of 23 ± 6 s.d. females (range: 15 to 32 females).

#### (ii) Samburu Elephant Project in Kenya

African savanna elephants have nested, multilayered (hierarchically structured), fission-fusion societies [39]. Associations vary with resource availability: smaller, single matrilineal groups are more common during dryer, resource-scarce periods, whereas larger herds of several hundred individuals can occur when resources are abundant [40]. Social learning is common in elephant societies with behaviours like crop-raiding learned from older individuals [41]. We used data on 201 female elephants from social networks with a mean of 116 ± 17 s.d. females (range: 97 to 130 females) from the Samburu Elephant Project in Kenya’s Samburu National Reserve collected between 1998 and 2014 (for observation methods see [42]; figure 1).

#### (iii) Shark Bay Dolphin Research Project in Australia

Indo-Pacific bottlenose dolphins are found in coastal waters throughout the western Pacific and Indian Oceans. These gregarious animals have regionally variable social dynamics, with individual-based fission-fusion behaviour being a key characteristic (see [43]), and display matrilineally transmitted cultural behaviours including the use of marine sponges as foraging tools in a subset of the population [44,45]. We used behavioural data from juvenile (i.e. weaned individuals, typically 4−10 years of age) and adult female dolphins (>10 years) from the Shark Bay Dolphin Research Project in Shark Bay, Australia [46] collected during boat-based surveys between 2008 and 2019 comprised of 178 unique females with networks averaging 132 ± 18 s.d. females (range: 102 to 150 females; figure 1).

### (b) Network construction for cross-species comparisons

To facilitate cross-species network comparisons, we accounted for variable data collection and different life histories by dividing the data into discrete ‘waves’ of one, two or three-year intervals for baboons, dolphins and elephants, respectively (electronic supplementary material, table S1). This allowed us to normalize lifespan differences and encompass the timescale necessary to capture temporal variation within groups. Within each wave, species-specific associations or affiliative interactions between individuals were represented as undirected, symmetric ties (see the electronic supplementary material). To control for sex-based social segregation [43], we focused only on social associations among females in each social network. Among mammals, females often play a central role in passing on social and ecological information to offspring and other group members [47]. More generally, the processes influencing social learning in males and females are expected to differ because of sexually dimorphic social constraints and, therefore, require sex-specific study [48]. Our work also extends previous studies focused on male removals (e.g. via trophy hunting [49,50]). Therefore, understanding the effects of female removals should offer important insights into the conservation of animal behavioural repertoires.

To quantify the effects of various individual removal scenarios on group social-cultural transmission, we (i) described network structural features for each species, (ii) tested the efficiency of within-wave information diffusion as a simple proxy for information transmission, and (iii) assessed group performance by simulating more biologically nuanced scenarios of information transformation via individual social learning and innovation. For step two, we compared the undisturbed group to random, age-based and key position-based removal scenarios. For step three, we compared the undisturbed group to random and age-based removal scenarios. All analyses were done in R v. 4.1.2 [51].

### (c) Modelling network structural features

We modelled independently weighted social networks for each species and wave using the *igraph* package [52]. In these networks, ‘nodes’ represent individuals that associate with each other via ties; interactions between individuals are weighted by interaction frequency (species-specific details in electronic supplementary material). From each network, we extracted individual-level degree and betweenness centrality, which indicate an individual’s importance in a group for facilitating information transmission. We then calculated five network structural features that could influence information transmission: modularity, transitivity, density, average degree (number of nodes) and average weighted path length (table 1) [30,53].

**Table 1.** Average network metrics (± s.d.) across all data waves for baboons, dolphins and elephants. No. nodes—number of individuals in a network. No. edges—the number of connections between members of the same network, defined as proximity or grooming within a society. Modularity—the tendency of a social unit to partition into subgroups of preferential associates. Transitivity—the proportion of triads (trios of three individuals) that all have three edges divided by the number of triads with two edges offers insights into the extent of connectedness among trios within social networks. Density—number of ties between individuals compared to how many ties could exist. Weighted path length—average degree of separation between individuals in a social network weighted by the number of individuals in a network.

species	no. waves	no. nodes	no. edges	modularity	transitivity	density	weighted path length
baboon	6	22.5 (±5.9)	112.8 (±39.8)	0.029 (±0.018)	0.740 (±0.038)	0.481 (±0.126)	0.028 (±0.010)
dolphin	6	132.3 (±17.5)	795.7 (±223.7)	0.429 (±0.042)	0.577 (±0.028)	0.090 (±0.013)	0.304 (±0.056)
elephant	3	115.67 (±16.9)	4920 (±959.2)	0.032 (±0.009)	0.852 (±0.023)	0.739 (±0.064)	0.019 (±0.001)

### (d) Efficiency as a proxy for information transmission

To conceptualize the capacity of each animal group to socially transmit information via member contact networks, we examined the efficiency of potential information spread between members. Here, efficiency is inversely proportional to the weighted path length between nodes and describes the potential for between-node communication [54]. This pairwise definition of efficiency can be expanded to describe the overall network efficiency as the sum of all inverse path lengths between node pairs. Efficiency will be higher in a network with shorter path lengths between nodes. The removal of individuals could affect the average length of paths in the network, thus affecting the structure’s ability to transmit information.

We first evaluated the potential efficiency of information diffusion for each wave of our three species’ networks (i.e. no removals). We then examined the effects of several types of individual removal— random, age-based and key position-based (degree or betweenness centrality)—on potential efficiency. Random removal served as both a baseline to understand the impact of any type of removal and a way to understand resilience to opportunistic hunting. Age-based removal maps most closely to expectations of natural death, including the possibility that the oldest individuals are most vulnerable to disease and to trait-based hunting (e.g. for elephant tusks). We also modelled the effects of losing individuals occupying key network positions (e.g. high degree, betweenness centrality) on group efficiency.

When simulating group member loss, we used a stepwise approach, removing 1% of nodes per wave at a time. This gradual removal of individuals continued until 20% of individuals were removed. Then, to assess the impact of these removals on the network’s efficiency, we compared network efficiency at each removal step to that of the intact network. Specific to random removal, we repeated the process of removal and efficiency calculation 100 times to account for the inherent variability resulting from random removals [55]. Finally, the efficiency values from the repeated random removal simulations were averaged to provide a more robust representation of the network’s efficiency throughout the removal process.

### (e) Social learning and innovation on fitness landscapes

We next evaluated groups’ ability to solve problems by comparing simulations of individual social learning and innovation before and after random and age-based removals. We used this approach to provide a more nuanced understanding of social learning across different innovation levels to acknowledge that exposure to social partners does not ensure social learning and to explore transmission of cultural variants of different utility. Specifically, we (i) used an NK model, which simulates a theoretical rugged fitness landscape where higher ‘elevation’ represents innovation of more advantageous behaviours or acquisition of information leading to increased fitness outcomes. In the model structure, we (ii) included age-based rules to account for three potential biological scenarios: certain individuals are more likely to innovate than others, older individuals have more successful behaviours (e.g. acquiring resources, accessing social benefits, etc.), and individuals learn via age-ranked transmission (i.e. from older individuals, not exclusively from parents). (iii) We used a range of innovation probabilities for each removal type to account for variable innovation rates. (iv) Lastly, the simulations included a series of timesteps wherein individuals explored the fitness landscape (innovate) and observed each other (socially learn). At each step, individuals (agents), only moved to a position—learned either socially or through innovation—if it was higher (more advantageous) in the fitness landscape. The simulations, then, reflect how the species’ networks can find the peaks in the rugged landscape, indicating learning that improves fitness, and how this search is potentially affected by varying innovation rates and different types of removal (see the electronic supplementary material).

#### (i) Simulating social learning strategies on fitness landscapes

We simulated problem-solving tasks to evaluate how social learning strategies of individuals influence group performance on a rugged fitness landscape. Since their introduction, rugged landscapes have been widely used to represent complex problem spaces [56] and examine how individuals within specific social structures can collaborate to solve such problems [57]. Within the landscape, higher positions represent solutions with increased fitness pay-offs (i.e. a better solution). Here, we generated a rugged fitness landscape (i.e. with multiple adaptive peaks, each with a local optimum) using an NK model [57]. In this landscape, *N* refers to the number of elements in a solution, whereas *K* refers to the interrelationships among these elements. Together, these were used to define the structure of the problem-solving task and the fitness payoff of each solution by including 2*^N^* potential positions in the fitness landscape and *K* ruggedness. In the simplest case for NK models (*K* = 0), there is a single peak; in the most complex case (*K* = *N* - 1), the landscape is dominated by multiple uncorrelated peaks. In other words, when *K* is low, the outcomes of innovation are straightforward—the height of the current position is highly predictive of the benefits to be gained in adjacent positions. When *K* is high, however, positions are uncorrelated, so each step might entail substantial changes in height. This could lead individuals to a local maximum rather than an overall optimal solution, thus making new strategies risky. We created a moderately complex landscape with *K* = 6 and selected *n* = 12 positions in the landscape. This yielded 4096 potential positions available to individuals in the network—many more possible positions than individuals in the network (see the electronic supplementary material).

#### (ii) Social transmission across innovation scenarios

We created a series of biologically based rules for how transmission of information within each innovation scenario can occur. Potential starting positions, equal to the number of individuals per wave, were randomly selected from the 4096 potential fitness landscape positions. Older individuals were placed in higher-quality positions, reflecting the assumption that age correlates with beneficial behaviours linked to survival. While high starting positions confer initial advantages, they could also result in individuals getting stuck at local optima because their positions are higher than the immediate vicinity but not the high points in the whole landscape. Such a pattern reflects a biological possibility where older, moderately successful individuals are less likely to seek newer, better strategies (e.g. dolphin tool use [58]). Additionally, we stipulated several rules for the social transmission of information via vertical learning. Specifically, individuals could (i) learn from better-positioned, older ties in their network at (ii) a probability equal to the relative weight of their tie to (iii) increase their fitness outcome from the acquisition of this knowledge [14]. For example, dolphin tool use is vertically transmitted from mother to primarily female offspring via social learning [59] with putative individual fitness consequences [60]. Thus, we assumed individuals would only move to a new position (through social learning) if this represented an increase in fitness. The rules above were applied to three currently debated innovation scenarios: younger individuals are more likely to innovate (young innovate) [61], older individuals are more likely to innovate (old innovate) [62] or both young and old innovate (all innovate) [63]. For the innovation scenarios, we categorized individuals into two age classes, ‘younger’ and ‘older,’ based on whether they were below or above the species' maximum peak reproductive age (baboons = 14 years, elephants = 40 years, dolphins = 25 years) [58,64,65].

#### (iii) Modelling how innovation rates moderate the effects of removals

For each innovation scenario, we tested how innovation rates, modelled as the probability of innovation π, moderate the effects of removal. At low values of π, indicating little innovation for new strategies, we expected age-based removal to harm the networks as there is little learning, making the accumulated knowledge (higher starting positions) of older individuals more important. At higher probabilities of innovation, however, older individuals that cannot learn might lower the average group performance of a population that continuously innovates. We explored values of π from 0 to 0.2 in increments of 0.02. These values reflect an extreme range of potential proportions of an individual’s time spent innovating, while acknowledging that—due to the error-prone nature of innovation—social learning remains the dominant strategy. For each value of π within each removal scenario, we ran 100 simulations.

#### (iv) Simulating the effects of age-based innovation on fitness

We ran 100 simulations at each innovation rate (enough time for the group response to reach an asymptote). At the start of each simulation, individuals were seeded following the rules for vertical transmission (see §2e(ii)). Then, for each removal scenario, no individuals (undisturbed), a random 10% of individuals (random removal) or 10% of the oldest individuals (age-based removal) were removed. We chose 10% removal because it doubles the average annual adult mortality for baboons [66], dolphins [67] and elephants [68], which we would expect to have a significant impact on each population. The model was then run for 500 timesteps, at the end of which the average fitness (height of all individuals) of the wave was recorded. Within each age-based innovation scenario, models adhered to the following rules at each timestep: (i) ‘innovators’ had π probability of exploring one adjacent position. (ii) However, individuals that explored an adjacent position only moved to that new position if doing so increased their fitness payoff. (iii) Although ‘non-innovators’ were not permitted to explore adjacent positions, if they were directly connected to at least one individual older than themselves then they could increase their fitness via social learning if they observed a position higher than their current one. Non-innovators socially learned from their older connection with the best position with a probability equal to the weight of their tie. (iv) Thus, the fitness of non-innovators also never decreased across time steps but could increase for those individuals directly connected to better-connected, older individuals (i.e. if they learned via transmission of information directly from associates older than themselves).

## 3. Results

### (a) Social network structural features

Although between-species statistical comparisons of network structures remain a major challenge in the study of animal social networks [30], our simulations offer preliminary insights into network features for each of the three species at multiple time points. Across waves (*n* = 6), baboon networks had an average of 23 (±5 s.d.) individuals and 113 (±40 s.d.) ties (table 1; electronic supplementary material, figure S1). While baboon networks were the smallest of the three species, their transitivity, density and average path length were between those of dolphin and elephant networks. Elephant networks had an average of 116 (±14 s.d.) individuals across waves (*n* = 3) and 4920 (±959 s.d.) ties. The considerable number of ties relative to individuals is likely due to large social groups formed during the wet season when resources are abundant [40,69]. Of the three species, elephant networks tended to have the most ties, be the densest and the most transitive, and, finally, have the smallest average path length between the individuals. Lastly, dolphin networks had an average of 132 (±16 s.d.) individuals across waves (*n* = 6) and 796 (±224 s.d.) ties. Their networks were the least dense, least transitive, most modular and had the largest average path length between individuals.

### (b) Efficiency as a proxy for information transmission

**Figure 2.**
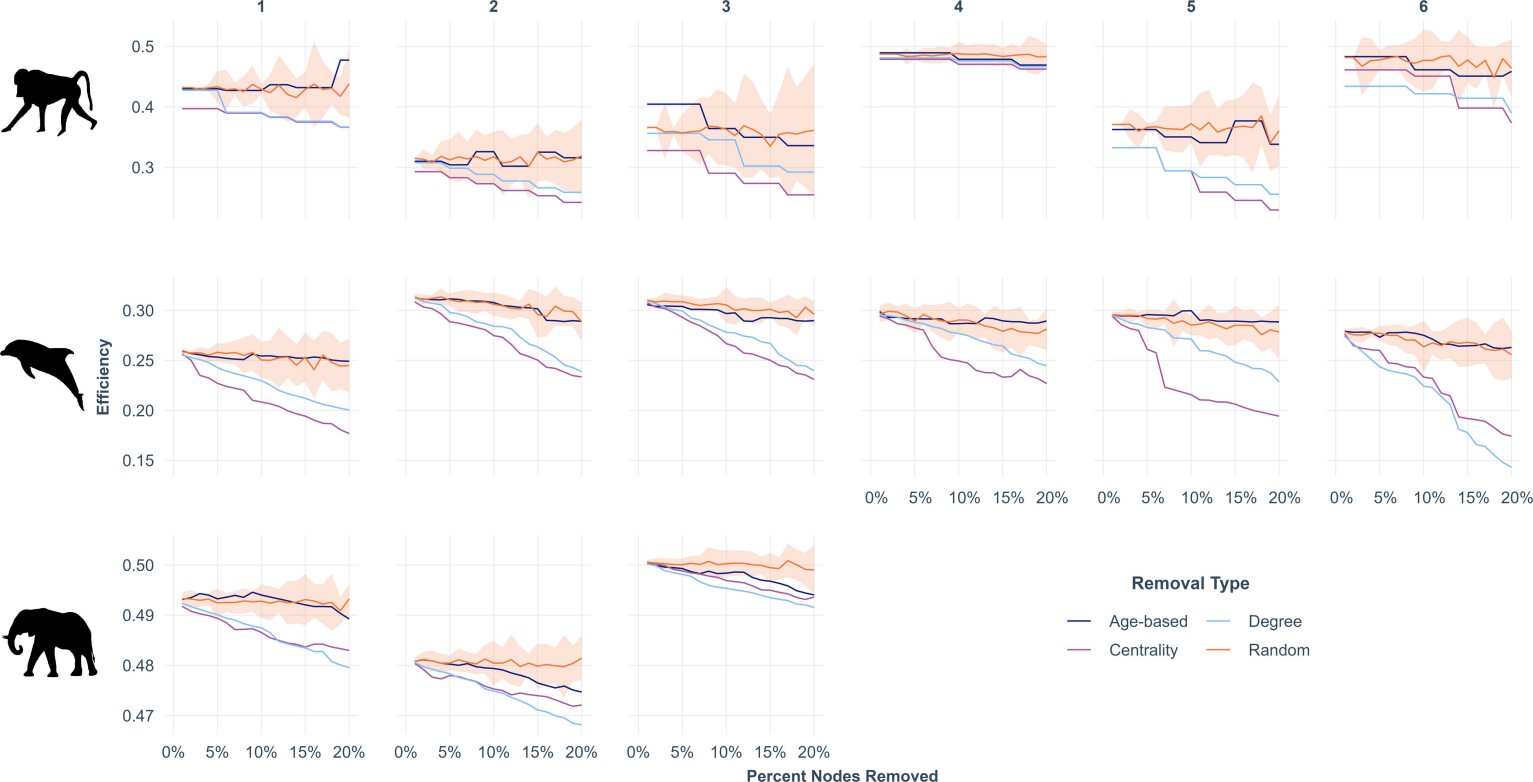
Global efficiency after removals. The plot shows the trajectories of global efficiency after a certain percentage of nodes (*x*-axis) are removed per wave (columns). Note variable *y*-axis per species. Orange ribbons represent the range of values produced by 100-random removal.

Prior to individual removal, the potential efficiency of the baboon, elephant and dolphin networks averaged 0.401 (±0.069 s.d.), 0.493 (±0.010 s.d.) and 0.298 (±0.069 s.d.), respectively across waves (figure 2). Age-based and random removals had a similar near-zero effect on efficiency (figure 2). Removal of key individuals, measured by degree or betweenness centrality, reduced group efficiency more than random or age-based removals (figure 2). In general, the baboon and dolphin networks displayed a greater efficiency loss after removals than the elephant networks.

### (c) Simulating information transmission

We simulated information transmission under three innovation scenarios: (i) both young and old individuals innovate (all innovate), (ii) only young individuals innovate (young innovate), and (iii) only old individuals innovate (old innovate). Here, we focus on the results of the all-innovate scenario. We then share case-specific results from the young innovate and old innovate analyses when they differ from the all-innovate scenario. Results of each simulation timestep are reported as the difference in average group fitness between the no-removal baseline and removal scenario. Negative values indicate reduced fitness relative to no removal scenarios. Conversely, positive values represent higher overall fitness compared to the scenario without any removals.

#### (i) All innovate scenario

**Figure 3.**
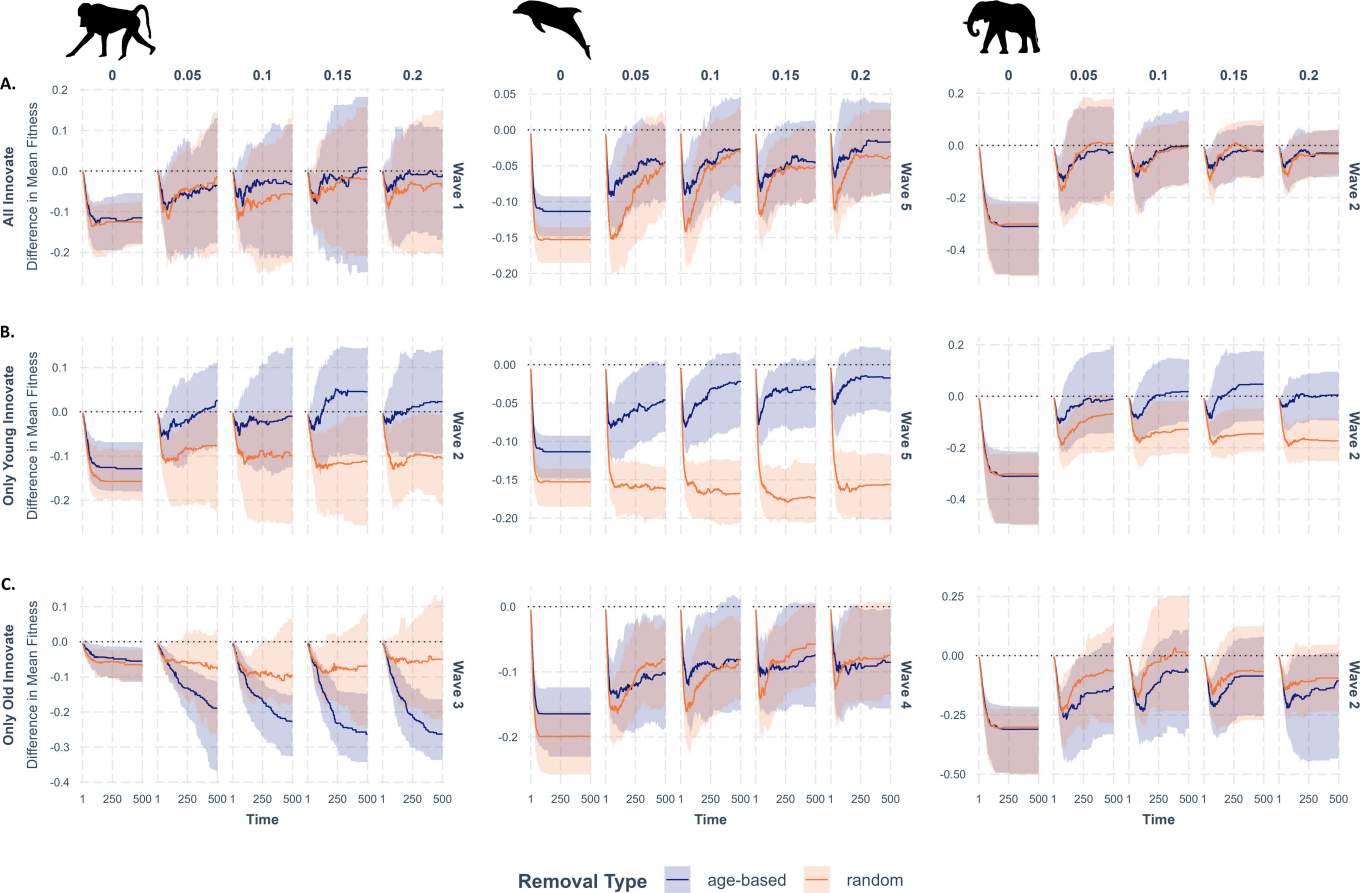
Example trajectories of differences in removal (random or age-based) from baseline fitness in cultural transmission simulations (A–C). Each row along the *y*-axis represents different innovator scenarios with variable ranges in the *y*-axis across species (major columns). Within each innovator scenario for a given species, the sequential plots represent examples of increased innovation rates from 0 to 0.2, and the simulation timesteps 1−500 within each innovation rate. The darker line represents the mean difference in fitness from non-removal for random removal (orange line) and age-based removal (purple line). Ribbon represents the 25th−75th quantile of the distribution.

In most waves across species, when all individuals innovate, both random and age-based removal resulted in a negative difference in mean fitness (i.e. reduced fitness, on average; subset in figure 3; full results in electronic supplementary material, figure S2). For innovation rates greater than zero, this negative difference is characterized by a steep initial decrease in fitness. In addition, across timesteps within and across rates of group innovation (π), both random and age-based removal showed similar decreasing gaps in fitness compared to no removal. For both removal scenarios, when the rate of innovation was low, removal tended to have a greater negative effect on average fitness. As innovation rate increased, the difference in fitness between removal and non-removal scenarios approached zero.

We found species-specific trends in the difference in mean fitness from the non-removal baseline following random and age-based removal (figure 3A). When all individuals innovated, the initial difference in mean fitness between removal and non-removal scenarios and recovery across timesteps was similarly small for baboons and elephants. After completion of all timesteps, removals had negligible fitness consequences for elephants and baboons, and the differences in fitness for both age-based and random removal scenarios generally reached zero for the simulations. The fitness gap did not close as quickly or become as small across timesteps and innovation rates for dolphins.

#### (ii) Young innovate scenario

The most notable difference between the young innovate and all innovate scenarios is the wider gap between random and age-based removal across timesteps and innovation rates (figure 3B). Unlike the all innovate scenario, the young innovate scenario with random removal consistently generated greater negative differences in mean fitness (i.e. reduced fitness) than age-based removals. Contrasting the other innovation scenarios for elephants, waves wherein young innovate generally maintained the greatest negative difference in mean fitness between random removal and non-removal and the greatest positive difference in mean fitness between age-based removal and non-removal across timesteps and rates of innovation. In some dolphin waves under the young innovate scenario, random removal increased negative differences in mean fitness across timesteps.

#### (iii) Old innovate scenario

Under the old innovate scenario, age-based removal generated negative differences in fitness greater or equal to that of random removal (figure 3C). This was particularly true for baboons where age-based removal generated increasingly negative differences in mean fitness with greater rates of innovation. For baboons and dolphins, greater rates of innovation generally had less effect on the difference in mean fitness compared to young innovate and all innovate scenarios.

## 4. Discussion

### (a) Network structural features vary with social cohesion

We used long-term data for three focal species to construct networks representing three levels of female social cohesion in animal social groups. We then derived commonly used network properties to determine the features most influential for social learning and information transmission. We found several major differences in the underlying network features across species and waves, which we attribute to variable species social dynamics.

First, across seasons, elephant networks were large and dense with many connections between individuals rather than separate subgroups. This elephant network structure was likely facilitated by the abundance of wet season resources, which can promote social cohesion [39]. In contrast, resource scarcity promotes preferential associations with kin, leading to hierarchical structures that result in periods of clustered elephant subgroups [70]. Dolphin networks were the least dense, least transitive, most modular and had the longest average path length between individuals. These dispersed networks with clustered subgroups resulted from individual dolphins temporarily separating from groups to avoid foraging or social competition [71,72]. Baboon networks were the smallest, but their transitivity, density, and average path length were in between those measures for dolphin and elephant networks. Because of their constant proximity and low social tolerance, baboon preferential affiliations are likely responsible for these structural features [73].

### (b) Network efficiency harmed by loss of key network positions

We calculated network efficiency before and after individual removals [54]. Before removals, elephant networks had the highest efficiency of the three species, indicating that information transmission is likely more effective and resilient in elephant networks than in the other study species. We attribute this, in part, to the high density and low modularity of elephant networks aggregated across seasons. Rather than a few subgroup-spanning individuals creating pinch points for information diffusion, individuals were connected through multiple pathways across seasons, creating more redundancy (resilience) for information flow. Elephants’ high mobility, which reduced observation frequency, necessitated aggregation of association data at a coarser temporal scale than dolphins and baboons. This likely inflated elephant network density calculations, which could explain why removals had a smaller effect on network efficiency for elephants than baboons and dolphins. That said, high-density aggregations in the wet season are characteristic of elephant social behaviour and explain high network efficiency for elephant networks [69].

Despite living in permanent social groups, baboon waves had moderate network efficiency relative to elephants and dolphins. This efficiency may reflect baboons’ low social tolerance, which results in distinct preferences for social partners with whom they share information [3,73]. Consequently, most learning and information transmission occurs through preferred affiliates [74], especially among younger individuals [75]. Despite this, we expected some learning from all individuals in the group as they are often in visual proximity. This affiliate-specific information pipeline results in little or no flow of information across subgroups. As such, it is important to note that our values for baboon network efficiency may reflect the biological reality of low social tolerance as well as the practical need for a more nuanced proximity metric that accounts for permanent group residence.

All three species experienced the greatest reduction in network efficiency following the removal of individuals in key network positions as compared to random and age-based removals. Despite removing up to 20% of individuals for random and age-based removals, efficiency stayed relatively constant, indicating that social groups are generally robust to the loss of older or less socially central individuals. This pattern underscores that important roles are not always linked to biological traits but are part of a complex interplay between network position and social influence in collective decisions [29,76–78]. Importantly, the network structure underlying group member affiliations may be key for ensuring the resilience of information transmission in the face of anthropogenic removal. More generally, it will be important to understand whether individuals with important network positions are also more vulnerable to human-induced mortality, and, if so, the extent to which these costs could outweigh positive links between sociality and longevity across taxa in many social mammals (e.g. killer whales [79], baboons [3], elephants [80]).

Overall, the loss of a greater number of individuals led to a greater decrease in efficiency; however, the severity of the decrease in their networks’ efficiency varied by species and their network structural features. Although methodological advances are needed to explicitly examine inter-specific differences in network characteristics influencing information transfer, our data offer preliminary evidence for each of the focal populations in this study. From a structural perspective, networks with low density, high modularity and long average path lengths (i.e. dense connections within subgroups but sparse inter-connections between them) are particularly susceptible to individual removal. For example, dolphin groups, which have the highest average path length, lowest density and high modularity across waves, consistently had the greatest loss of efficiency with the removal of individuals in key network positions. Compared to dolphins, baboons and elephants have similar levels of modularity, but the elephant group was denser, with more ties between individuals, which made it easier for elephants to maintain capacity for transmission through interconnected network structure despite individual loss.

The resilience of elephant and baboon efficiency (following individual loss) seen in our study aligns with previous observations connecting the role of key network positions and network structure to cultural resilience in these species. Strong kin-biased associations among elephants [81] may contribute to the ability of elephants to maintain the transfer of information despite loss of individuals. For example, if an elephant matriarch dies, her equally well-connected daughter replaces her in the group [82]. Whereas if an important female baboon dies, multiple individuals with weaker ties collectively compensate for her loss [83]. In contrast, dolphins’ foraging innovations primarily transmit vertically through matrilines with some behaviours exhibited by only one female and her descendants [84]. Consequently, the loss of one individual could eradicate a particular foraging tactic. For example, dolphins that use sponge tools represent only 5% of the population [85], so the removal of a few individuals could mean the loss of the behaviour. Moreover, because foraging is usually a solitary activity, there is a low probability of transmission except within matrilines, especially for rare tactics [85]. The apparent role of low modularity in maintaining network efficiency provides a starting point for a networks-based approach identifying groups of a species that are most vulnerable to loss of cultural variants.

### (c) Innovation recovers fitness benefits of social learning

To go beyond efficiency, a static estimate of social learning, we simulated generic problem-solving in a theoretical ‘rugged landscape’ and compared group responses in removal and non-removal scenarios. Across age-based and random removals, removals universally reduced mean fitness regardless of species, innovation scenario or innovation probability. Although the loss of individuals initially fractured network connections and reduced opportunities for social learning, innovation was the driving force behind recovery of group fitness over time. In other words, greater innovation appeared to increase exploration for previously unknown solutions with higher fitness pay-offs over favouring known solutions with lower pay-offs stemming from older individuals [86]. In almost all waves, increasing the rate of innovation led to an improvement in overall group fitness because novel solutions could emerge that were not inhibited by broken network connections or by older individuals teaching suboptimal problem-solving techniques. However, in situations where there were no or very few individuals capable of innovating, increasing the innovation rate did not improve fitness and, in some cases, widened the gap in fitness between removal and non-removal groups over time.

Without the introduction of innovations, connected individuals quickly transferred existing information, leaving the difference in group fitness static. The growing gap between baseline and removal groups, where essentially one group is innovating and the other is not, highlights the potential value of innovation in group recovery after disturbance. While the role of innovation in facilitating group recovery after disturbance has not yet been studied, our findings are in line with evidence suggesting that innovation leading to behavioural diversity enables species to exploit new niches [87]. Nonetheless, although our results are consistent with theoretical and empirical expectations of social learning, our models were not designed to fully capture the potential costs of ‘bad’ innovations and future work should do so.

Removal had a disproportionately negative effect on group fitness when removal targeted the innovator group. In waves where all individuals innovated, we saw similar trends of a shrinking gap in mean fitness for both removal scenarios because innovators were removed in both scenarios. This mirrors our findings regarding the effects of removal on efficiency, whereby random and age-based removal had a similarly small effect. The effect of random removal on group fitness, when only young individuals innovated, was greater than the effect of age-based removal on group fitness when only old individuals explored. These findings inform recent debates [61–63] about the importance of young versus older individuals in innovating solutions.

## 5. Implications for culture conservation

Across species, many groups experience losses that start with the gradual reduction of group size and result in increased risk for an ‘extinction spiral’ [88]. Given the increasing threat of human-driven removals of animals from their social groups, it is crucial to consider and implement effective interventions that can support resilience in animal societies to reduce further negative fitness consequences. Although social animals have evolved adaptive responses to cope with the loss of specific associates [83,89], the current magnitude of anthropogenic impact is outpacing evolved responses [24], resulting in the disruption of social groups. Our study demonstrates the negative effects of these removals across multiple taxa, but also suggests that social groups can display a surprising amount of resilience to loss, especially when the loss follows a predictable pattern, such as the loss of old individuals, and when rates of innovation are high. These findings extend previous research describing several adaptations to the loss of group members, including group policing to enforce diverse interactions between group members or equally well-connected offspring compensating after the loss of a parent [82,90]. More broadly, these patterns underscore the emerging idea that animal social structures, including social connections [32] and social information [91]—that together contribute to animal culture [11]—can play a vital role in the conservation of social species.

## Data Availability

The dolphin data and code necessary for reproducing this analysis are available at https://github.com/NicolasRestrep/diffusion_animal_networks. The baboon data for the analysis can be accessed at [92] and the elephant data can be accessed at [93]. Supplementary material is available online [94].
